# Adverse events and tolerability of long-term suppressive antibiotic therapy for periprosthetic joint infection: a prospective cohort study

**DOI:** 10.5194/jbji-11-267-2026

**Published:** 2026-05-12

**Authors:** Pia Reinecke, Svetlana Karbysheva, Stavros Goumenos, Anna Conen, Olga Pidgaiska, Carsten Perka, Andrej Trampuz, Thilo Khakzad, Sebastian Meller

**Affiliations:** 1 Charité – Universitätsmedizin Berlin, Centre for Musculoskeletal Surgery, corporate member of Freie Universität Berlin, Humboldt-Universitaät zu Berlin, and Berlin Institute of Health, Berlin, Germany; 2 Clinic for Infectious Diseases and Infection Prevention, Kantonsspital Aarau, Aarau, Switzerland; 3 School of Clinical Sciences, Faculty of Health, Queensland University of Technology, Brisbane, Queensland, Australia; 4 Royal Brisbane and Women's Hospital, Herston, Queensland, Australia

## Abstract

**Background**: Long-term suppressive antibiotic therapy (SAT) is a management strategy for periprosthetic joint infection (PJI) when eradication is unattainable. However, SAT is often associated with adverse events (AEs) that may impact quality of life. This study aimed to evaluate the prevalence and types of AEs, assess patient-reported tolerability, and identify patient- and antibiotic-related factors associated with AEs during SAT for PJI. **Methods**: A retrospective analysis of a prospectively established cohort of 30 patients receiving SAT for PJI following DAIR (Debridement, Antibiotics, and Implant Retention) or prosthesis exchange between 2019 and 2022 was conducted at a specialized septic surgery department. SAT was indicated for suboptimal surgery or absence of biofilm-active antibiotics and was administered for ≥6 months. Structured patient interviews and institutional records were used to capture AEs, treatment modifications, SAT discontinuation, infection-related complications, and subjective tolerability. Patients were stratified by sex, age, BMI, and antibiotic agent. **Results**: The median SAT duration was 55 weeks (interquartile range (IQR): 40–101). Non-serious AEs occurred in 97 % of patients ( n=29 ), predominantly affecting the gastrointestinal tract ( n=63 ), skin ( n=35 ), and nervous system ( n=25 ). Treatment modifications were required in 23 % of patients, most frequently with amoxicillin or cotrimoxazole. SAT discontinuation occurred in one patient (3 %) due to multiple AEs. Four patients (13 %) experienced SAEs related to persistent or recurrent infection rather than antibiotic toxicity. Median subjective tolerability was 8 out of 10 (IQR 6–8); 57 % of patients rated tolerability as “good”, 37 % as “moderate”, and 6 % as “poor”. **Conclusion**: SAT for PJI is associated with a high prevalence of mild to moderate AEs, but treatment discontinuation is rare. Most patients report acceptable tolerability, and serious antibiotic-related complications are uncommon. Careful patient selection, individualized monitoring, and management of AEs are essential to optimize long-term outcomes.

## 1 Introduction

Periprosthetic joint infection (PJI) is a serious complication in orthopaedic surgery, occurring in up to 16.4 % of hip and knee revision arthroplasties and carrying a 3-year mortality rate of 18.8 % in patients undergoing revision for infection (Patel, 2023; Grimberg, 2023). Bacterial colonization of prostheses leads to biofilm formation, which complicates eradication and typically requires prolonged therapy with biofilm-active antibiotics. Certain pathogens, such as *Streptococci* spp., lack effective biofilm-active agents (Renz et al., 2019), and the presence of antibiotic-resistant bacteria, often classified as difficult-to-treat (DTT) pathogens, further limits therapeutic options and is associated with poorer outcomes (Margaryan et al., 2025).

Additionally, a subset of patients is unable or unwilling to undergo revision surgery due to comorbidities or surgical risk, precluding definitive infection control (Burr et al., 2022). In these cases, suppressive antibiotic therapy (SAT) offers a strategy to control infection; prevent acute systemic deterioration (e.g., sepsis); and limit local complications such as abscesses, fistulas, or prosthesis loosening while preserving the implant (Burr et al., 2022; Malahias et al., 2020; Wouthuyzen-Bakker et al., 2017). SAT is typically tailored to the causative pathogen, yet definitions and treatment protocols remain inconsistent across institutions and literature (Patel, 2023).

Prolonged SAT, typically lasting months to years, commonly employs amoxicillin, cotrimoxazole, and tetracyclines (Keller et al., 2016; Prendki et al., 2014, 2017; Sandiford et al., 2020; Pradier et al., 2017, 2018). Nevertheless, extended antibiotic use carries an increased risk of adverse events (AEs), including gastrointestinal disturbances and hypersensitivity reactions, with severity influenced by both drug and patient factors (Escudero-Sánchez et al., 2020; Escudero-Sanchez et al., 2020; Keller et al., 2016; Pradier et al., 2017; Pradier et al., 2018; Prendki et al., 2017; Wouthuyzen-Bakker et al., 2017). Long-term therapies can also impair quality of life, contribute to chronic side effects, and increase the risk of antibiotic resistance (Bindel and Seifert, 2025; Li et al., 2020).

Despite the growing use of SAT, data on AE prevalence, characteristics, and patient-reported tolerability remain limited. With the rising incidence of PJI and the expanding role of SAT (Wengler et al., 2014; Kurtz et al., 2010), better understanding these factors is essential to optimize therapy and improve outcomes.

This study aims to (1) determine the prevalence and characteristics of AEs during SAT following revision surgery for PJI, (2) assess patient-reported SAT tolerability, and (3) explore the impact of various patient- and antibiotic-related factors on AE occurrence.

## 2 Materials and methods

### 2.1 Study design

After institutional board approval (no. EA4/040/14), a prospectively established cohort of patients who received SAT for PJI in our specialized septic surgery department at a single academic institution between January 2019 and December 2022 was retrospectively analysed. PJI was diagnosed according to European Bone and Joint Infection Society (EBJIS) criteria and managed by an interdisciplinary musculoskeletal infection team (Karczewski et al., 2019; McNally et al., 2021; Patel, 2023; Renz and Trampuz, 2023).

Exclusion criteria were an age under 18 years, language barriers, loss to follow-up, ongoing antibacterial therapy for non-PJI, SAT duration < 6 months, incomplete records, or missed reimplantation in planned multi-stage exchange.

Antimicrobial therapy followed published guidelines, consisting of initial intravenous treatment followed by oral therapy for up to 12 weeks. SAT was defined as oral antibiotics continued beyond 12 weeks for ≥6 months or for a lifelong period. Dosing followed protocol and was adjusted for renal/hepatic function and treatment duration (Renz et al., 2019; Renz and Trampuz, 2023; Sandiford et al., 2020; Sandiford and Granger, 2020).

SAT was indicated when (1) surgery was suboptimal or not feasible (e.g., incomplete implant removal, excessive comorbidity or anesthetic risk) or (2) no biofilm-active agent was available (e.g., rifampicin-resistant staphylococci, ciprofloxacin-resistant Gram-negative bacteria, streptococci or fungi), necessitating prolonged therapy to prevent relapse and local complications (Sandiford and Granger, 2020; Renz and Trampuz, 2023).

### 2.2 Definitions of AEs

AEs related to SAT were defined as newly observed, drug-related side-effects that are well-established as antibiotic AEs and that occurred within the expected time frame after initiation of therapy. These definitions were derived from previous publications, institutional standards, and consensus opinions (Escudero-Sánchez et al., 2020a, b; Keller et al., 2016; Leijtens et al., 2017, 2019; Nguyen et al., 2015; Pradier et al., 2017., 2018; Prendki et al., 2017; Shah et al., 2020; Tonnelier et al., 2021; Wouthuyzen-Bakker et al., 2017; Edwards and Aronson, 2000).

AEs attributable to other concurrent chronic medical treatments were not considered. A serious adverse event (SAE) was defined as an untoward medical occurrence which required inpatient hospitalization, was life-threatening, resulted in a significant disability or incapacity, or led to death. Non-serious AEs were defined as medical occurrences that did not meet SAE criteria and did not trigger emergency alerts (Table S1 in the Supplement).

### 2.3 Data collection

Data were collected through structured interviews conducted during clinical visits or by telephone. Each patient was interviewed once, either at the conclusion of SAT or at least 6 months after its initiation. Interview timing varied according to follow-up duration, calculated as the interval between SAT initiation and the interview date. Standardized questionnaires (S2) were used to capture AEs previously described in the literature (Sandiford et al., 2020; Escudero-Sánchez et al., 2020a, b; Keller et al., 2016; Leijtens et al., 2017, 2019; Pradier et al., 2017, 2018; Prendki et al., 2017; Shah et al., 2020; Tonnelier et al., 2021) and were supplemented by institutional records. The overall number and types of AEs, treatment modifications, and infection-related complications requiring hospitalization or revision surgery were recorded. Subjective tolerability was assessed using a numerical scale from 0 to 10 (0 = very poor tolerance; 10 = good tolerance) and was subsequently categorized as “good”, “moderate”, or “poor”. Additional patient-reported information, including emotional impact, treatment experiences, and adherence, was documented. Clinical data including age, body mass index (BMI), Charlson comorbidity index (CCI), surgical strategy, antimicrobial agents used for SAT, and causative pathogens were extracted from medical records

### 2.4 Statistical analyses

The prevalence of AEs and SAT tolerability were analysed on a per-patient basis. Patients were stratified by sex, age ( <65 years vs. >65 years), BMI ( <25 g m^−2^ vs. >25 kg m^−2^) (Sweatt et al., 2024), and antibiotic used for SAT (amoxicillin, doxycycline, or cotrimoxazole). AE prevalence across organ systems was compared between groups. Continuous variables are presented as medians with interquartile ranges (IQRs), and categorical variables are presented as absolute counts and percentages. Group differences were assessed using the Mann–Whitney U test for continuous variables and the $\chi^{2}$ or Fisher's exact tests for categorical variables, as appropriate. Comparisons involving more than two groups were conducted using the Kruskal–Wallis test. Due to the limited sample size, multivariable analyses were not performed. All tests were two-sided, with p values < 0.05 considered to be significant. Statistical analyses were performed using IBM^®^ SPSS^®^ Statistics (Version 29).

## 3 Results

### 3.1 Patient characteristics

**Figure 1:**
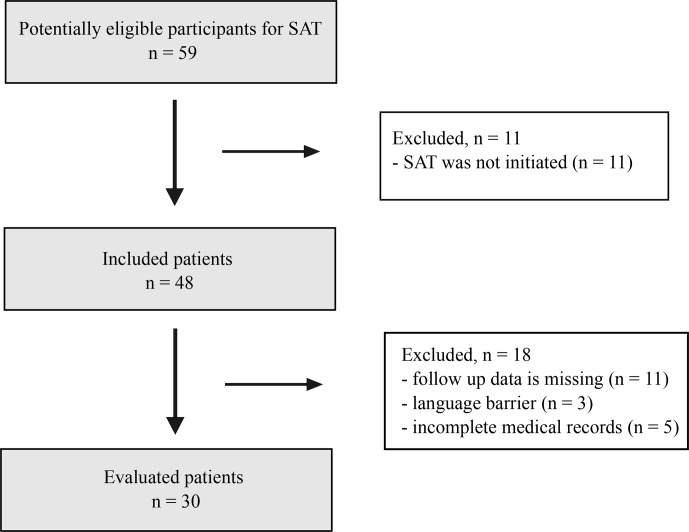
Flowchart of patient selection.

A total of 59 patients were screened for eligibility. After exclusions, 30 patients were included in the final analysis (Fig. 1).

The primary surgical strategy consisted of two-stage prosthesis exchange in 27 patients (90 %) and a DAIR procedure in 3 patients (10 %). Polymicrobial infections were present in 9 patients (30 %). The most common causing pathogens were *Streptococcus* spp. in 17 patients (57 %). Detailed patient characteristics are presented in Tables 1 and 2.

**Table 1:** Demographic data and infection characteristics of 30 patients.

Characteristics	Patients ( n=30 )
Male, n (%)	15 (50)
Median age, years (IQR)	72 (64–80)
≥65 years, n (%)	24 (80)
Median BMI (IQR)	26 (23–29)
BMI ≥ 25 kg m^−2^	17 (57)
Affected joint, n (%)	
Hip	25 (83)
Knee	5 (17)
Median CCI (IQR)	4 (3–5)
Comorbidities, n (%)	
Hypertension	18 (60)
Diabetes mellitus	4 (13)
Liver cirrhosis	2 (7)
COPD	3 (10)
Malignancy	5 (17)
Rheumatoid arthritis	1 (3)
Chronic kidney disease	10 (33)
Antibiotics used for SAT, n (%)	
Amoxicillin	15 (50)
Doxycycline	9 (30)
Cotrimoxazole	6 (20)
Metronidazole	1 (1)

IQR – interquartile range; BMI – body mass index; CCI – Carlson comorbidity index; SAT – suppressive antibiotic therapy; COPD – chronic obstructive pulmonary disease.

### 3.2 Antibiotic treatment

The prescribed antibiotics, causative pathogens, and duration of SAT are summarized in Table 2. The median duration of SAT was 55 weeks (IQR: 40–101). Treatment duration varied and was guided by the clinical course of PJI, antibiotic tolerability, and individual patient preferences.

### 3.3 Adverse events and associated risk factors

The median follow-up was 32 months (IQR: 23–39). A total of 145 non-serious AEs were recorded during SAT. Overall, 29 of 30 patients (97 %) experienced at least one non-serious AE, whereas 1 patient (3 %), treated with doxycycline for rifampicin-resistant *Staphylococcus epidermidis*, reported none. A total of 19 patients (63 %) experienced 5 or fewer AEs, 10 (33 %) had 5 to 10, and 1 (3 %) reported 24 AEs. The gastrointestinal tract was the most frequently affected organ system ( n=63 ), with diarrhea, reflux, and nausea predominating, followed by skin and skin appendage disorders ( n=35 ), mainly rash, pruritus, and dry skin. Neurological AEs ( n=25 ) included fatigue, neuropathy or paresthesia, and depressive symptoms. Other commonly reported events included weight change and myalgia or arthralgia (Tables S2 and S3). Treatment modifications, including dose reductions or antibiotic switches, were most frequently associated with amoxicillin and cotrimoxazole. SAT discontinuation occurred in one patient (3 %) receiving cotrimoxazole due to multiple AEs.

AE rates did not differ significantly by sex or age. Patients with BMI ≥ 25 kg m^−2^ showed a higher overall AE burden compared with patients with BMI < 25 kg m^−2^, approaching statistical significance ( p=0.054 ). Kidney-related AEs were more frequent with cotrimoxazole than with amoxicillin or doxycycline ( p=0.033 ). No other antibiotic-specific differences were observed.

**Table 2:** Characteristics of 30 patients and isolated microorganisms with corresponding SAT.

Patient	Pathogen^a^	Antibiotic (daily dose)	Duration (weeks)	FU^b^ (months)	AE/SAE	SAT status^c^/ adjustments
1	*E. cloacae*, *S. epidermidis* (MRSE)^1^	Cotrimoxazole 3×960 mg	29	45	Liver/revision	complete
2	*S. mitis/oralis* ^2^	Amoxicillin 3×1 g	53	44	GI;PCNS/–	complete/–
3	*S. epidermidis* (MRSE)^1^	Doxycycline 2×100 mg	50	36	none	complete/–
4	*S. epidermidis* (MRSE)^1^	Doxycycline 2×100 mg	55	42	GI/–	complete/–
5	*S. warneri* ^1^	Doxycycline 2×100 mg	169	47	GI; SA/–	complete/–
6	*E. coli*, *E. cloacae*, *S. aureus* (MSSA)^3^	Cotrimoxazole 1×960 mg	95	38	GI; kidney/–	ongoing/dose reduction
7	*S. agalacticae* ^2^	Amoxicillin 3×1 g	155	36	All/–	ongoing/dose reduction
8	*S. mitis/oralis* ^2^	Amoxicillin 3×1 g	30	34	GI/–	complete/switch
9	*C. difficile* ^4^	Metronidazole 3×400 mg	84	31	GI/revision	complete
10	*S. dysgalacticae* ^2^	Cotrimoxazole 2×480 mg	47	44	GI; SA; kidney/–	complete/–
11	*S. mitis/oralis* ^2^	Amoxicillin 3×1 g	200	46	SA/–	complete/–
12	*S. dysgalactiae* ^2^	Amoxicillin 3×1 g	41	25	GI; SA; PCNS; kidney/–	complete/switch
13	*S. mitis/oralis* ^2^	Amoxicillin 3×1 g	156	47	GI; SA; other/–	complete/switch
14	*S. dysgalactiae* ^2^, *P. mirabilis*, *E. coli* (MDR)^5^	Cotrimoxazole 3×960 mg, Amoxicillin 3x1g	24	21	GI; PCNS; BL;other/–	complete/discont.
15	*S. dysgalactiae* ^2^	Amoxicillin 2×1 g	150	31	SA; PCNS; other/–	complete/–
16	*S. haemolyticus* ^1^	Doxycycline 2×100 mg	51	29	GI; SA; PCNS; BL; other/–	complete/–
17	*S. dysgalactiae* ^2^	Amoxicillin 3×1 g	104	22	GI; SA; PCNS/revision	ongoing/dose red
18	*P. miribalis* ^5^	Doxycycline 2×100 mg	39	19	GI; SA; BL/revision	ongoing/–
19	*S. epidermidis* (MRSE)^1^, *E. cloacae* ^5^	Cotrimoxazole 2×960 mg	25	6	GI; SA; kidney/–	complete/–
20	*S. epidermidis* (MRSE)^1^, *S. aureus* (MSSA)	Doxycycline 2×100 mg	31	28	GI; SA/–	complete/–
21	*S. mutans* ^2^	Amoxicillin 3×1 g	69	17	GI/–	ongoing/dose red

**Table 2:** Continued.

Patient	Pathogen^a^	Antibiotic (daily dose)	Duration (weeks)	FU^b^ (months)	AE/SAE	SAT status^c^/ adjustments
22	*S. agalactiae* ^2^	Amoxicillin 3×1 g	77	18	GI; SA; PCNS; other/–	ongoing/–
23	*S. agalactiae* ^2^	Amoxicillin 3×1 g	55	38	GI; PCNS/–	ongoing/–
24	*E. coli* ^5^, *S. aureus* (MSSA), *S. epidermidis* (MRSE)^1^	Cotrimoxazole 3×960 mg	28	36	GI; SA; PCNS; BL; liver/–	complete/–
25	*S. agalactiae* ^2^	Amoxicillin 3×1 g	52	35	GI; SA; PCNS, other/–	complete/–
26	*S. aureus* (MSSA)^3^	Doxycycline 2×100 mg	104	23	GI; SA; PCNS, kidney/–	ongoing/–
27	*S. agalactiae* ^2^, *K. pneumoniae*	Amoxicillin 1×1 g	67	29	GI; PCNS/–	ongoing/–
28	*S. aureus* (MSSA), *P. aeruginosa* ^5^	Doxycycline 2×100 mg	100	23	GI; SA; PCNS/–	ongoing/–
29	*S. dysgalactiae* ^2^	Amoxicillin 3×1 g	55	33	GI; SA/–	complete/–
30	*S. mitis/oralis* ^2^, *P. saccharolyticus*, *E. faecalis*	Doxycycline 2×100 mg	54	27	GI/–	complete/–

MDR – multi drug resistance; MSSA – methicillin-susceptible *S. aureus*; MRSE – methicillin-resistant *S. epidermidis*; GI – gastrointestinal tract; SA – skin and skin appendages; PCNS – peripheral and central nervous system; BL – bone marrow/blood; – means SAT remained unchanged. ^a^ Reason for SAT: ^1^ rifampicin-resistent staphylococci, ^2^ Streptococci, ^3^ suboptimal surgery, ^4^ no biofilm active agent, ^5^ ciprofloxacin-resistent Gram-negative bacteria. ^b^ Time from initiation of SAT to the interview. ^c^ By time of the interview.

Four patients (13 %) experienced SAEs requiring inpatient hospitalization. Two underwent revision during ongoing SAT – one for acute culture-negative PJI on amoxicillin-based SAT and one for persistent infection with a newly identified pathogen on doxycycline-based SAT. Two additional patients required revision after completion of SAT: one due to recurrent infection with the index pathogen following metronidazole-based SAT and one due to infection with a newly identified pathogen after cotrimoxazole-based SAT (Table 2).

### 3.4 SAT tolerability

Subjective quantitative tolerability assessments of SAT were available for all patients. The median tolerability rating was 8 points (IQR: 6–8). Qualitative evaluation revealed that 57 % of patients ( n=17 ) rated SAT tolerability as “good”, with more male ( n=10 ) than female patients ( n=7 ) providing this rating. A total of 11 patients (37 %) reported “moderate” tolerability (7 male, 4 female), while 2 patients (6 %) categorized tolerability as “poor” (1 female, 1 male). At the time of interviewing, 20 patients (67 %) had completed SAT, while 10 patients (33 %) were still receiving ongoing therapy. Patients who completed SAT reported that AEs persisted throughout treatment but resolved rapidly after discontinuation.

## 4 Discussion

This study demonstrates that AEs during SAT are common but generally do not affect adherence as only 1 of 30 patients discontinued treatment. Overall, SAT was well tolerated, with most participants reporting good or moderate tolerability. While previous research has largely focused on the efficacy of SAT, few studies have systematically assessed tolerability or captured patient-reported experiences. To our knowledge, this is the first study to comprehensively document all AEs encountered during SAT for PJI using the most frequently prescribed antibiotics for this indication and incorporating subjective feedback.

The high prevalence of AEs in our cohort (97 %) likely reflects the detailed assessment through structured interviews, exceeding previously reported rates of 14 %–53 % (Escudero-Sánchez et al., 2020a, b; Keller et al., 2016; Leijtens et al., 2019; Malahias et al., 2020; Nguyen et al., 2015; Pradier et al., 2017, 2018; Prendki et al., 2017; Shah et al., 2020; Tonnelier et al., 2021; Wouthuyzen-Bakker et al., 2017). Despite this, the pattern of AEs aligns with previous findings: gastrointestinal symptoms were more frequent, followed by dermatological manifestations. Bone-marrow-related AEs were infrequent, potentially underreported due to limited laboratory monitoring during routine outpatient care. Nevertheless, the frequency observed aligns with the existing literature (Escudero-Sánchez et al., 2020a, b; Keller et al., 2016; Leijtens et al., 2019; Malahias et al., 2020; Nguyen et al., 2015; Pradier et al., 2017, 2018; Premkumar et al., 2021; Prendki et al., 2017; Shah et al., 2020; Tonnelier et al., 2021; Wouthuyzen-Bakker et al., 2017). Cotrimoxazole use was associated with a higher frequency of renal AEs ( p=0.033 ), consistently with known nephrotoxic effects (Fraser et al., 2012; Li et al., 2019). However, this does not necessarily indicate true nephrotoxicity or reduced glomerular filtration as direct measures of kidney function were not performed. The finding should be interpreted cautiously since cotrimoxazole can raise serum creatinine by inhibiting tubular secretion without reflecting an actual decline in renal function. Treatment adjustments were required in 23 % of patients, most often involving dosage modifications or antibiotic switches. SAT discontinuation remained rare, underscoring the importance of balancing AE management with the therapeutic goal of infection suppression. These findings emphasize individualizing SAT, particularly in patients with multiple comorbidities or extensive antibiotic exposure.

The absence of standardized guidelines for SAT duration reinforces the necessity of a tailored approach. In our cohort, SAT ranged from 24 weeks to 4 years, with a recommended minimum of 6 months and longer when tolerated. Lifelong SAT may be required when eradication is unfeasible, such as in the case of persistent infection, compromised soft tissue or bone, or unmodifiable risk factors. SAT is especially valuable for elderly patients and those with megaprostheses who are poor candidates for complex surgical revision (Pilge et al., 2012; Janz et al., 2020; Prendki et al., 2017; Burr et al., 2022; Malahias et al., 2020; Leijtens et al., 2019; Sandiford et al., 2020; Siqueira et al., 2015; Renz et al., 2019). Our indications for SAT align with the current literature and clinical practice (Sandiford et al., 2020; Sandiford and Granger, 2020).

Consistently with previous studies, amoxicillin was among the most frequently used antibiotic in our cohort, reflecting its favourable tolerability profile and activity against common PJI pathogens such as *Streptococcus* spp., *Enterococcus* spp., and *Cutibacterium* spp. (Sandiford et al., 2020; Keller et al., 2016; Prendki et al., 2014, 2017; Fröschen et al., 2022). Oral tetracyclines are a reliable alternative in selected cases (Pradier et al., 2017, 2018).

While most AEs were non-serious, four patients experienced SAEs, two during ongoing SAT. Importantly, these events were related to persistent or recurrent infection requiring revision surgery rather than antibiotic toxicity, underscoring the fact that SAT is a non-curative, palliative strategy. No SAEs were attributable to life-threatening antibiotic toxicity, permanent disability, or death, indicating that severe treatment-limiting complications from antibiotics are uncommon and typically reflect the complexity of the patient population. High treatment adherence likely reflects patients' perception of SAT as essential for maintaining joint function and avoiding further surgery. All patients who completed SAT reported that AEs resolved shortly after treatment cessation, suggesting that chronic toxicity may be less limiting than anticipated. These observations are consistent with prior reports indicating that SAT-related AEs are generally reversible (Schindler et al., 2013; Shah et al., 2020).

Recent findings from Nandi et al. (2024) further support the favourable tolerability of SAT without clear evidence of increased resistance among causative PJI pathogens (Nandi et al., 2024).

Collectively, these findings, together with comparable success rates reported elsewhere, reinforce SAT as a valid management strategy in carefully selected PJI cases, particularly those caused by streptococci or antibiotic-resistant difficult-to-treat pathogens.

This study has several limitations. Its single-centre design and relatively small sample size limit generalizability and preclude multivariable analyses. Tolerability assessments were based on patient interviews conducted without a standardized time frame, and subjective attribution of AEs introduces inherent bias. Data on antibiotic resistance were not systematically collected, and validated patient-reported outcome measures were not used, limiting a more granular evaluation of quality-of-life impact and the balance between clinical benefit and harm.

## 5 Conclusion

SAT for PJI was associated with a high prevalence of AEs; however, these were predominantly mild to moderate, rarely led to treatment discontinuation, and seldom resulted in serious antibiotic-related complications. Most patients reported acceptable tolerability and remained adherent despite persistent symptoms, suggesting that perceived benefits of infection suppression and joint preservation outweigh associated harms in carefully selected individuals. SAEs were uncommon and primarily related to infection persistence or progression rather than antibiotic toxicity, underscoring the palliative nature of SAT.

Both antibiotic- and patient-related factors, particularly cotrimoxazole use and higher BMI, influenced AE occurrence, highlighting the importance of individualized treatment and close monitoring.

Overall, SAT represents a feasible management option when curative surgical treatment is not achievable. Careful patient selection, ongoing tolerability assessment, and timely reassessment of surgical options are essential. Future multi-centre studies incorporating standardized outcome measures and patient-reported outcomes are needed to better define the risk profile of SAT and optimize individualized treatment strategies.

## Supplement

10.5194/jbji-11-267-2026-supplement The supplement related to this article is available online at https://doi.org/10.5194/jbji-11-267-2026-supplement.

## Data Availability

The data supporting this study originate from our institutional software and personal interviews. All data are maintained in accordance with institutional data protection and privacy standards; therefore, they are not publicly available.
