# Supplementary material for: Adverse events and tolerability of long-term suppressive antibiotic therapy for periprosthetic joint infection: a prospective cohort study
Source: J Bone Jt Infect. 2026 May 12;11(3):267–75. doi: 10.5194/jbji-11-267-2026 (PMC13163188; doi:10.5194/jbji-11-267-2026)
Supplement: The supplement related to this article is available online at https://doi.org/10.5194/jbji-11-267-2026-supplement. [file jbji-11-267-2026-supplement.zip › jbji-11-267-2026-supplement-title-page.pdf]

Supplement of J. Bone Joint Infect., 11, 267–275, 2026
https://doi.org/10.5194/jbji-11-267-2026-supplement
© Author(s) 2026. CC BY 4.0 License.

Supplement of

Adverse events and tolerability of long-term suppressive antibiotic therapy for periprosthetic joint infection: a prospective cohort study

Pia Reinecke et al.

Correspondence to: Sebastian Meller (sebastian.meller@charite.de)

- jbji-11-267-2026-supplement-title-page.pdf
- JBJI_Supplement_S1_Tables.pdf
- JBJI_Supplement_S2_Questionnaire.pdf

The copyright of individual parts of the supplement might differ from the article licence.
