# Supplementary material for: Adverse events and tolerability of long-term suppressive antibiotic therapy for periprosthetic joint infection: a prospective cohort study
Source: J Bone Jt Infect. 2026 May 12;11(3):267–75. doi: 10.5194/jbji-11-267-2026 (PMC13163188; doi:10.5194/jbji-11-267-2026)
Supplement: The supplement related to this article is available online at https://doi.org/10.5194/jbji-11-267-2026-supplement. [file jbji-11-267-2026-supplement.zip › JBJI_Supplement_S1_Tables.pdf]

Supplement S1

Table S1. Definitions of adverse events

**Non-serious adverse event**	**Definition**
C. difficile infection	Clinical signs and symptoms consistent with a *C. difficile* infection in the setting of a positive *C. difficile* toxin PCR test result from stool
Anemia	Hemoglobin level < 10 g/dL at least 10 days after last surgery
Leukopenia	White blood cell count < 4500 leukocytes/L
Thrombocytopenia	Platelet count < 150.000/L
Renal failure	Increase in serum creatinine level > 1.5 time the patients' baseline
Hepatitis	Increase in aspartate transaminase or alanine transaminase level > 3 times the patients' baseline
Changes in weight	Patients' subjective impression of losing or gaining weight during the time of antibiotic treatment
Changes in electrocardiogram	Prolonged QTc-time; > 550 ms
**Serious adverse event**	**Definition**
Inpatient hospitalization	Any initial admission to a hospital or equivalent health care facility, even if for less than 24 hours; any prolongation of an existing admission
Life-threatening	AE which places the patient at immediate risk of death at the time of the event
Significant disability/incapacity	A substantial disruption of a person's ability to conduct normal life functions
Death	Death related to SAT

Table S2. Frequency of AE stratified by the involved organ systems

Organ system	n (%)[1]	Adverse Events	n (%)[2]
Gastrointestinal tract	63 (42)	Diarrhea	12 (19)
		Reflux	8 (13)
		Nausea	8 (13)
		Inappetence	7(11)
		Dry mouth	5 (8)
		Dysgeusia	5 (8)
		Constipation	4 (6)
		Clostridioides difficile enterocolitis	4 (6)
		Vomiting	3 (5)
		Meteorism	3 (5)
		Malaise	2 (3)
		Change in color or texture of the tongue	2 (3)
Skin and skin appendages	35 (23)	Skin rash	10 (29)
		Pruritus	8 (23)
		Dry skin	7 (20)
		Candida mucositis (oral and genital)	5 (14)
		Skin swelling	3 (9)
		Changes in hair or nails	1 (3)
		Photosensitivity	1(3)
Peripheral and central nervous system	25 (17)	Fatigue	4 (16)
		Neuropathy, paresthesia	4 (16)
		Symptoms of depression	4 (16)
		Vertigo	3 (12)
		Visual problems	3 (12)
		Headache	3 (12)
		Memory issues	3 (12)
		Dizziness	1 (4)
Kidney	6 (4)	Renal failure	6 (100)
Bone marrow/blood	5 (3)	Anemia	4 (80)
		Leukopenia	1 (20)
Liver	3 (2)	Hepatitis	3 (100)
Other	12 (8)	Weight gain	4 (33)
		Weight loss	3 (25)
		Myalgia and/or arthralgia	3 (25)
		Dyspnea	1 (8)
		Changes in electrocardiogram (long QTc)	1 (8)

[1] refer to number of all adverse events (n=149)

[2] refer to number of AE within the organ system

Table S3. Frequency of AE stratified according to sex, age, BMI and antibiotic used for SAT

Organ system	Female (n=15)	Male (n=15)	p-value	Age < 65 years (n=7)	Age ≥ 65 years (n=23)	p-value	BMI < 25 kg/m² (n=13)	BMI ≥ 25 kg/m² (n=17)	p-value	Amoxicillin (n=15)	Doxycycline (n=9)	Cotrimoxazole (n=6)	p-value
**Gastrointestinal tract (n = 63)**	28 (45)	35 (55)	0.431	15 (24)	48 (76)	0.562	24 (38)	39 (62)	0.355	23 (37)	26 (41)	9 (14)	0.615
**Skin and skin appendages (n = 35)**	14 (40)	21 (60)	0.316	5 (14)	30 (86)	0.129	9 (26)	26 (74)	0.068	14 (40)	14 (40)	6 (17)	0.941
**Bone marrow and blood (n = 5)**	2 (40)	3 (60)	0.777	0	5 (100)	0.184	2 (40)	3 (60)	0.871	2 (40)	2 (40)	1 (20)	0.847
**Liver (n = 3)**	1 (33)	2 (67)	0.550	1 (33)	2 (67)	0.671	0	3 (100)	0.116	1 (33)	0	2 (67)	0.055
**Kidney (n = 6)**	1 (17)	5 (83)	0.073	1 (17)	5 (83)	0.671	1 (17)	5 (83)	0.147	2 (33)	1 (17)	3 (50)	0.033
**Peripheral or central nervous system (n = 25)**	8 (32)	17 (68)	0.206	3 (12)	22 (88)	0.586	7 (28)	18 (72)	0.377	14 (56)	7 (28)	4 (16)	0.254
**Other[1] (n = 12)**	3 (25)	9 (75)	0.334	2 (17)	10 (83)	0.506	3 (25)	9 (75)	0.588	9 (75)	2 (17)	1 (8)	0.256
**All adverse events (n = 145)**	57 38)	92 (62)	0.241	27 (18)	122 (82)	0.536	46 (31)	103 (69)	0.054	65 (44)	52 (35)	26 (17)	0.976

Data are shown as number of AEs (%) within this group.

BMI, body mass index

[1] weight gain, weight loss, myalgia and/or arthralgia, dyspnea, changes in electrocardiogram (long QTc)
