# Supplementary material for: Adverse events and tolerability of long-term suppressive antibiotic therapy for periprosthetic joint infection: a prospective cohort study
Source: J Bone Jt Infect. 2026 May 12;11(3):267–75. doi: 10.5194/jbji-11-267-2026 (PMC13163188; doi:10.5194/jbji-11-267-2026)
Supplement: The supplement related to this article is available online at https://doi.org/10.5194/jbji-11-267-2026-supplement. [file jbji-11-267-2026-supplement.zip › JBJI_Supplement_S2_Questionnaire.pdf]

Supplement S2

Study Questionnaire

Charité Centrum für Orthopädie und Unfallchirurgie

Charité | Centrum für Muskuloskeletale Chirurgie
Campus Virchow-Klinikum | Augustenburger Platz 1 | 13353 Berlin

Centrum für Muskuloskeletale Chirurgie

Ärztlicher Direktor: Univ.-Prof. Dr. med. Carsten Perka
Geschäftsführender Direktor: Univ.-Prof. Dr. med. Ulrich Stöckle

Campus Virchow-Klinikum, Augustenburger Platz 1, 13353 Berlin
Service-Hotline: Tel. +49 30 450 552 688, Fax +49 30 450 755 2977
Privatsprechstunde: Tel. +49 30 450 552 032, Fax +49 30 450 552 901
E-Mail: cmsc-virchow@charite.de
Web: cmsc.charite.de

Patientenfragebogen - Unerwünschte Arzneimittelwirkungen während der Langzeitantibiotikatherapie

---

Name Vorname Patientennummer

Sind Sie sich vor Beginn der Therapie bewusst darüber gewesen, welche Nebenwirkungen auftreten könnten?

Ja ☐ Nein ☐

Sind Ihnen während der Therapie Veränderungen aufgefallen, die Sie in Zusammenhang mit der Antibiotikatherapie bringen?

Ja ☐ Nein ☐

Hatte die Therapie Auswirkungen auf bereits bestehende Vorerkrankungen?
Wenn Ja, welche?

Ja ☐ Nein ☐

Wurde die Therapie aufgrund von Nebenwirkungen unterbrochen?
Wenn Ja, wie lange?

Ja ☐ Nein ☐

Welche weiteren Maßnahmen wurden ergriffen, um die Nebenwirkungen zu behandeln?

Ergab sich danach eine Besserung?

Ja ☐ Nein ☐

Wie haben Sie die Therapie im Allgemeinen vertragen?

0 1 2 3 4 5 6 7 8 9 10

Therapie abgebrochen/ umgestellt — Sehr gut Keinerlei Beschwerden

Wie stark hat Sie die Therapie in Ihrem Alltag eingeschränkt?

Sehr stark — stark — teils teils — wenig — gar nicht

Hatten Sie nach Beginn bzw. Ende der antibiotischen Therapie einen weiteren ungeplanten Krankenhausaufenthalt im Rahmen der Therapie des entzündeten Gelenkes und gab es weitere Operationen?

Organsystem	Nebenwirkung	Nein	Ja	Anmerkungen
Gastrointestinal/ Magen Darm Trakt	Durchfall	☐	☐	
	Übelkeit	☐	☐	
	Erbrechen	☐	☐	
	Clostridium difficile Enterokolitis	☐	☐	
	Appetitlosigkeit	☐	☐	
	Verstopfungen	☐	☐	
	Aufgeblähtes Gefühl	☐	☐	
	Reflux	☐	☐	
	Unwohlsein	☐	☐	
	Veränderter Geschmack	☐	☐	
	Mundtrockenheit	☐	☐	
	Veränderung der Beschaffenheit oder Verfärbung der Zunge	☐	☐	
Haut	Ausschlag	☐	☐	
	Schwellungen	☐	☐	
	Juckreiz	☐	☐	
	Photosensibilität	☐	☐	
	Veränderung von Haut- oder Haarbeschaffenheit	☐	☐	
	Orale/ Vaginale Candida Mukositis	☐	☐	
	Trockene Haut	☐	☐	
Nervensystem	Fatigue (Müdigkeit)	☐	☐	
	Neuropathie, Parästhesie (Missempfindungen)	☐	☐	
	Symptome einer Depression	☐	☐	
	Schwindel	☐	☐	
	Sehstörungen	☐	☐	
	Kopfschmerzen	☐	☐	
	Gedächtnisstörungen	☐	☐	
	Verwirrtheit	☐	☐	
Niere	Niereninsuffizienz	☐	☐	
Hämatogen/ Blut	Anämie	☐	☐	
	Leukopenie	☐	☐	
Leber	Hepatitis (Leberfunktionsstörung)	☐	☐	
Sonstiges	Gewichtszunahme	☐	☐	
	Gewichtsverlust	☐	☐	
	Myalgie und/oder Arthralgie (Muskel-/ Gelenkschmerzen)	☐	☐	
	Dyspnoe (Kurzatmigkeit)	☐	☐	
	EKG-Veränderungen (QT-Zeit Verlängerung)	☐	☐	
